# Hospital and Health News

**Published:** 1923-06

**Authors:** 


### HOSPITAL AND HEALTH NEWS.

### The Registration of Herbalists.

Commander Kenworthy has presented a Bill for the registration of herbalists, which seeks to establish a general medical herbalists' council.

### A Hospital Presentation.

Mr. Arthur Bowdery has retired on a pension from the post of inspector at Hanwell Mental Hospital after thirty-three years' service in all grades. The officers and staff have presented him with a clock.

### Change of Name.

The City of London Hospital for Diseases of the Heart and Lungs is the new name by which the "Victoria Park Hospital" is to be known. The old name will be retained as a sub-title.

### A "Starr" Hospital.

Mr. S. M. Mitra, a Hindu, has suggested in the "Daily Telegraph" that a "Starr Hospital" should be established at Kohat, with Mrs. Starr as its superintendent, to commemorate her recent act of courage.

### Condensed Milk Regulations.

The Ministry of Health has issued Public Health (Condensed Milk) Regulations prescribing the labelling and composition of condensed milk. The Regulations will come into operation on October 1.

### The Prevention of Tuberculosis.

The annual conference of the National Association for the Prevention of Tuberculosis will be held at Birmingham on Thursday and Friday, July 12 and 13. The conference will be presided over by the Chairman of the Council, the Hon. Sir Arthur Stanley, and those who have promised to take part include Viscount Astor, Sir Henry Gauvain and Sir Gilbert Baring.

### Health Conference at San Francisco.

An International Health Education Office is to be held as a part of the World Conference on Education at Oakland, San Francisco, from June 28 to July 6. The purpose is to discuss aims and ideals, subject matter, and methods of health education in relation to all school-age children. The problem of training leaders and teachers in the field of health education will be given special consideration.

### Prizes in the Hospitals Competition.

A Bristol lady won the first prize, £7,500, in the competition in aid of the Hospitals of London Combined Appeal; a Southport lady the second prize, £2,000; and a Birmingham gentleman the third, £1,000. It is understood that there were over a quarter of a million competitors, and as the principal prizes were presented, and most of the expenses guaranteed, the competition has raised a large sum.

### The Ranyard Mission.

The annual meeting of the Ranyard Mission will be held on Friday, June 1, at 3 p.m., in the Central Hall (small), Westminster. The chair will be taken by the Bishop of Woolwich, and the speakers will be Lady Sydenham, Miss Irene M. Hett (Hon. Sec., Ranyard Mission), Mr. J. Prescott Hedley, F.R.C.S. (St. Thomas's Hospital), the Rev. Dr. Bardsley, and the Rev. Ivor J. Roberton. The sixty-sixth birthday gathering of the workers will be held immediately after the meeting.

### Worthing Hospital Extension Opened.

The Earl of Athlone has opened the new wing at Worthing Hospital, which includes wards for maternity and paying patients, and is the principal section of the extension scheme that had to be postponed a few years ago. In his speech Lord Athlone reminded the audience that the hospital's Secretary, Capt. A. O. Kaye, and himself had served in the same regiment, and it was through Mr. Kaye that he was present that day. Lord Athlone also presented the matron, Miss Ellen Burford, with an illuminated address and a cheque for £71 on her retirement after twenty-eight years.

### Castles as Hospitals.

Lord Londonderry has offered Seaham Hall, and Lord Boyne Brancepeth Castle to Durham County for use as hospitals. Both were used as hospitals or convalescent homes during the war. Brancepeth is said to have been originally built before the Conquest, and to be perhaps the oldest castle in the country. The Local Voluntary Hospitals Committee called the meeting at which the offer was considered. The offer of Brancepeth was reluctantly declined because the cost of alteration would be too heavy. The acceptance of Seaham Hall remains in the balance.

### The Late Sir John Holder

The recent death of Sir John Holder, Bart., at the age of 85, has deprived the General Hospital, Birmingham, and indeed most of the local charities, of a staunch friend and most generous benefactor. He received his baronetcy in 1897, shortly after the new General Hospital was opened, and there were very few of its original or subsequent needs to which he did not contribute his personal service and the aid of his purse. His name will always be associated with the hospitals and the universities of his native city.

### Hospital Fire at Brighton.

A serious fire at the War Hospital Supply Depot and the Princess Royal Red Cross Auxiliary Hospital for Officers, in Percival Terrace, Brighton, was with difficulty extinguished. It started between the second and third floor, and attacked four rooms occupied by patients, some of whom were helpless. Forty-three patients, all army officers, were in the hospital; all were rescued. The origin of the fire is unexplained.

### Another Insulin Discovery.

Dr. Best, who was associated with Dr. Banting in the discovery of insulin, and Dr. D. A. Scott, one of the directors of the Connaught Laboratories, have reported to the Toronto Academy of Medicine that an insulin-like substance has been found in the blood of animals. The substance seems to have many of the essential effects of the original extract. It markedly lowers the sugar in the blood stream and produces symptoms characteristic of an overdose of insulin. The practical value of the discovery is not yet determined.

### The Combined Hospitals Appeal.

The accounts of the Hospitals of London Combined Appeal are being rapidly wound up, and a balance-sheet and report will be presented to the Prince of Wales, President, and the General Council of King Edward's Hospital Fund for London, at a meeting to be held on June 4. The offices of the Appeal at 19 Berkeley Street, are being closed, and all communications relating to the Appeal should be addressed to Devonshire House, Piccadilly, W. The address of the Hospital Saving Association, the permanent organisation which has been set up to organise a contributory scheme for wage-earners, will be 77 Cambridge Terrace, W.2.

### The Central Joint V.A.D. Council.

The Army Council recently extended an invitation to the St. Andrew's Ambulance Association to nominate a representative to serve on the Central Joint V.A.D. Council. The Association has now nominated Colonel Donald J. Mackintosh, C.B., M.V.O., of the Western Infirmary, Glasgow, to serve in this capacity. This appointment practically completes the constitution of the Council, which is now representative of every important ambulance organisation in the country, and which has been sent up for the purpose of reorganising the V.A.D. organisation.

### A Medical Officer's Salary.

The Wrexham Rural District Council and the Wrexham Town Council recently offered in an advertisement a salary of £650, including travelling expenses, for a new joint medical officer of health. The Welsh Board of Health advised them that the figure would not be likely to guarantee satisfactory service from a whole-time officer, and they suggested for consideration of a more adequate commencing salary for such an important post. At a special meeting of the District Council it was unanimously decided to authorise their representatives, in their negotiations with the Town Council, to fix a commencing salary of £750, with two annual increments of £25. The Wrexham Town Council also discussed the question and agreed unanimously that £700 would be an adequate salary, this amount exceeding that which was paid to the late medical officer on his appointment in 1919.

### The Proposed School of Hygiene.

The Minister of Health, with the concurrence of the Trustees of the Rockefeller Foundation, has appointed a Transitional Executive Committee in connection with the proposed School of Hygiene. The functions of the committee will be to appoint a director to arrange for amalgamation or co-ordination between the school and other institutions working in similar or closely related spheres, to prepare plans for the new school, and to begin building, unless in the meantime it has been possible to set up the permanent governing body. The members of the committee are: Mr. Neville Chamberlain, M.P. (chairman), Lord Burnham, Captain Sir Arthur Clarke, K.B.E., C.B.E., Sir Walter Fletcher, K.B.E., M.D., F.R.S., Lieut.-Col. Fremantle, O.B.E., F.R.C.S., M.P., Sir Harry Goschen, K.B.E., Sir George Newman, K.C.B., M.D., Sir Cooper Perry, M.D., and Sir Arthur Robinson, K.C.B., C.B.E., with Mr. L. G. Brock, C.B., of the Ministry of Health, as secretary.

### Open-Air Nursery Schools.

In accordance with the resolution passed at the Islington Conference on Day Nursery Schools, reported in our last issue, a deputation approached the London County Council on the matter. The speakers were Miss Margaret McMillan, O.B.E., and Councillor the Rev. J. F. Matthews (Woolwich). The deputation was received by the Children's Care Sub-committee of the London County Council. Miss Margaret McMillan laid stress on the financial aspect of the matter, emphasising the importance of a day nursery school as a measure of economy. The cost per child per annum was about £14 3s. Referring to the question of sites, she stated that no attempt had been made to look for them, but no difficulty was anticipated; she was sure they could be obtained. The members asked a number of questions, and at the conclusion the Chairman stated that the Sub-committee would very carefully consider everything the deputation had put before them.

### Lectureship in Morbid Psychology.

An important development in the post-graduate work of the Faculty of Medicine at Birmingham University is announced. Since 1919 the University, through the generosity of Sir Charles Hyde, has had an annual lecture on psychotherapy. In conjunction with the Asylums Committee, a Board of Research has been set up upon which the Committee and the University are jointly represented. In order that that scheme might be carried out a Director of Mental Research, with a competent assistant, was required. The funds for the actual research work, which will be carried on at Hollymoor for the present, have been found by the Asylums Committee. The University share consists in altering both the title and the conditions of the lectureship in psychotherapy. With the consent of Sir Charles, the lectureship has been turned from an annual lectureship into a lectureship for three years in morbid psychology, which is now the title it bears. Sir Frederick Mott has been appointed Director of Research and Lecturer.

### Regional Hospital Committees.

At the meeting of the Regional Committee of the British Hospitals' Association held at Portsmouth Royal Infirmary, Mr. A. Godwin Pratt (Honorary Treasurer, Royal Victoria and West Hants Hospital, Bournemouth) was elected Chairman of the Committee and Mr. Gordon M. Saul (Secretary, Royal Victoria and West Hants Hospital) was appointed Honorary Secretary. In 1921 the British Hospitals' Association formed Regional Committees, the local Region including the hospitals at Southampton, Portsmouth, Winchester, Bournemouth, West Sussex, the Isle of Wight and the adjacent cottage hospitals. The meetings of the Regional Committee are held quarterly by invitation at the various hospitals, and much useful work has been accomplished in co-ordinating the work of the voluntary hospitals. Information was obtained and supplied and expert evidence given to the Voluntary Hospitals' Parliamentary Commissioners. Other matters undertaken by the Regional Committee, in conjunction with the parent Association, include negotiations with the Ministry of Pensions for the treatment of pensioners and, with the Approved Societies, to allocate a share of the surplus funds under the National Health Insurance Act. The exemption of nurses from the provisions of the Unemployment Insurance Act has also been obtained, and many other helpful and important matters affecting the voluntary hospitals accomplished.

### Extension of University College Hospital.

The King and Queen will pay a peculiarly interesting visit to University College Hospital and University College on May 31. The ceremony is in connection with the great gift made in 1921 by the Rockefeller Foundation of New York for medical education. It will have two features, the laying of the foundation stones of the new obstetric hospital and new nurses' home now being erected on sites adjacent to University College Hospital by Mr. Paul Waterhouse, F.R.I.B.A., and Mr. George Hornblower, F.R.I.B.A., and the opening of the new anatomy building which has already been erected in Gower Street, to the south of the main College buildings, from the designs of Professor F. M. Simpson, F.R.I.B.A. After the first part of the ceremony, their Majesties will proceed across Gower Street, and the King will declare open the anatomy building. The gift of the Rockefeller Foundation for these important medical objects is a sum of £400,000 for the erection of the buildings in connection with University College Hospital and Medical School; £435,000 for an endowment fund for the improvement of medical teaching and for the purpose of developing general medical education and research on modern lines; £370,000 for the erection and endowment of the anatomy buildings at University College, including the extension of the physiology and pharmacology buildings. The total benefaction thus amounts to £1,205,000, and is probably the largest single benefaction ever provided in this country for educational purposes.

---

### NURSES' REGISTRATION.

There are reasons for believing that there are still a considerable number of nurses who are either ignorant of the fact that the period of grace for the registration of existing nurses comes to an end on July 14 of the present year, or, not being ignorant, are under the impression that they will be able to register somehow or other even after the period of grace has expired.

#### Existing Nurses.

An "Existing Nurse" is, according to the Act, a nurse who was for at least three years before the first day of November, 1919, bona fide engaged in practice as a nurse in attendance on the sick under conditions which appear to the Council to be satisfactory for the purposes of the provision of the Act and has adequate knowledge and experience of the nursing of the sick. The rules made by the Council under this section of the Act are too lengthy for insertion here; they can be obtained at the office of the Council, 12 York Gate, Regent's Park. Any nurse eligible under these rules, who fails to apply for registration before July 14 next, will not be able to be registered except by passing the Council's examinations after having undergone a training of at least three years in a hospital approved by the Council. The first of the final or qualifying examinations will be held in July, 1925.

#### Intermediate Nurses.

These are nurses who began their training on or after November 1, 1916, that is to say, too late to complete a three years' period of nursing before November 1, 1919. These nurses will be able to register without undergoing the Council's examinations provided they apply for registration before July, 1925, and produce a certificate that they have had not less than three years' training before July, 1925, in a hospital, voluntary or Poor Law, approved by the Council. The foregoing statements refer to nurses who wish to register on the general part of the Register, but so far as the dates of the expiration of the periods of grace for existing and intermediate nurses are concerned, they apply also to nurses who desire to register on the supplementary parts of the Register for male, mental, sick children's and fever nurses.

